# Mercy killing of a 72‐year‐old woman through heroin intoxication

**DOI:** 10.1111/1556-4029.15637

**Published:** 2024-11-07

**Authors:** Johann Zwirner, Stefanie Iwersen‐Bergmann, Klaus Püschel, Benjamin Ondruschka

**Affiliations:** ^1^ Institute of Legal Medicine University Medical Center Hamburg‐Eppendorf Hamburg Germany; ^2^ Department of Oral Sciences University of Otago Dunedin New Zealand

**Keywords:** active euthanasia, alprazolam, external postmortem, heroin, hydroxyalprazolam, mercy killing

## Abstract

Active euthanasia is legally permissible in only eight jurisdictions worldwide and may only be administered by qualified personnel following specific selection criteria. Mercy killing refers to the deliberate termination of the life of an individual suffering from a terminal chronic medical condition. Detecting both illegally performed active euthanasia and instances of mercy killing presents challenges for forensic pathologists. The presented case describes a mercy killing involving a 72‐year‐old woman with multiple chronic conditions who was killed by her grandson via heroin intoxication after administration of the anxiolytic alprazolam. Key findings from the external postmortem examination included a single fresh injection site on the inside of the elbow and a superficial T‐shaped cut on the flexor side of the left forearm. Toxicological analyses revealed elevated blood levels of heroin metabolites, including 6‐monoacetylmorphine and absence of hydroxyalprazolam, indicating an only short survival time following heroin injection. A cocaine concentration in blood was comparatively low but rather high in hair samples. Elderly individuals with multiple chronic conditions are at increased risk of becoming homicide victims. Comprehensive forensic documentation of injection sites is essential to avoid overlooking deaths caused by injection and to differentiate them from medical measures during resuscitation attempts.

**Highlights**

- A rare mercy killing case of an elderly person through heroin injection is described.
- Thoroughly performed external postmortem investigations are mandatory.
- Elderly individuals with multiple chronic conditions are prone to become homicide victims.

## 1 INTRODUCTION

Active euthanasia refers to the deliberate killing of a terminally ill person by active means and has been legalized in only eight jurisdictions worldwide, with Portugal being the latest country to join the list in May 2023, pending implementation [1, 2]. However, from experience, a legal ban alone is insufficient to completely eliminate instances of active euthanasia. According to an anonymous online questionnaire conducted among 2507 physicians and 2683 nurses in Germany, participation in active euthanasia was reported in 1.94% and 1.24% of cases, respectively, within the preceding 24 months [3]. In countries where active euthanasia and assisted suicide (suicide assisted by another person) are legal, physicians are typically required to oversee the process [4]. Nevertheless, in certain instances, individuals known as “mercy killers,” such as family members, friends, or caregivers, may actively end the life of a suffering person with the intention of relieving their pain [1]. Therefore, mercy killing is a form of active euthanasia, which is illegal in all jurisdictions. In all cases of consensual homicides or voluntary euthanasia, conducting a thorough external postmortem investigation is crucial for detecting signs of foul play, being the basis for a subsequent autopsy including toxicological analysis and ultimately the solution of the case. Presented is a case of mercy killing, accompanied by discussions on the implications for forensic practice.

## 2 CASE REPORT

### 2.1 Crime scene investigation

**FIGURE 1:**
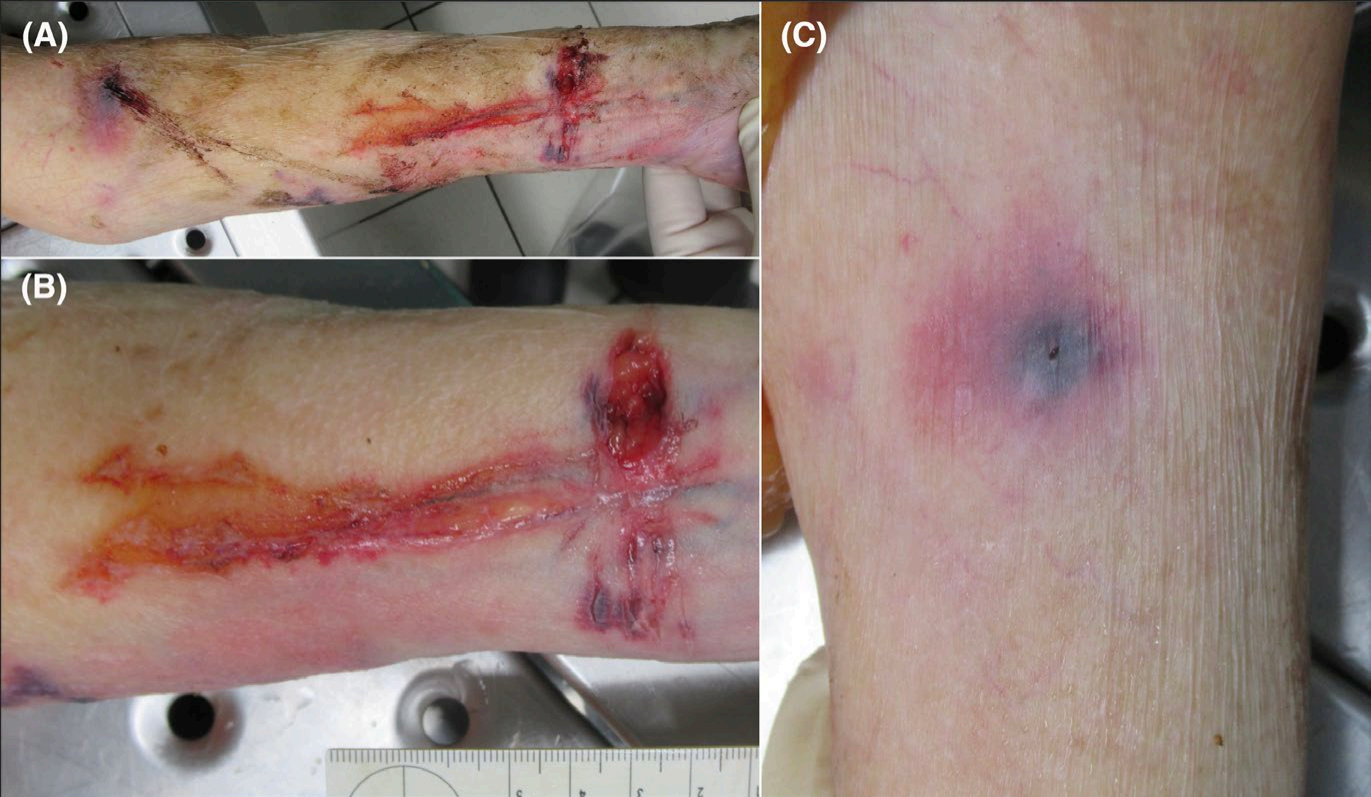
Autopsy findings. (A) The left forearm is shown before cleaning. The superficial T‐shaped incision (B) and the injection puncture and bruise (C) are shown after cleaning.

A 72‐year‐old woman was found lifeless in supine position covered with a blanket. She was dressed in regularly sitting thin clothes. Blood stains, partly wet, were observed at various locations: around the midpoint of the left clavicle, along the outer portion of the mattress to the left side of the body, and on the mattress underneath the left arm. An incision, superficial and forming a T‐shape, extended to the subcutaneous fat with hemorrhagic infiltration of the wound edges, was identified in the lower third of her left forearm's flexor side (Figure 1A,B). Both the stem and arm of the T‐shaped cut comprised multiple individual and superficial incisions with partial bleedings into the wound edges. The contralateral hand of the deceased exhibited no blood stains, and no potential cutting instrument was found neither in her right hand nor in closer proximity to the body. A punctate skin defect with surrounding hematoma and minimal discharge of partially dried blood from the center was observed in the left cubital fossa (Figure 1A,C). At scene, livor mortis was still blanchable upon pressure, and rigor mortis was absent in the mandibular joint but present in the elbow and knee joints. The rectal temperature was 29.7°C. Multiple blood stains, including passive and transfer blood stains, were scattered throughout the disordered flat. Unused butterfly needles and a recapped scalpel without apparent blood stains were found in the living room, while a single razor blade was discovered in a pool of blood within the hallway adjacent to the living room. Two handwritten notes were recovered: a farewell letter penned by the deceased expressing her desire to end her life and a note written by her grandson admitting to administering 6 mg alprazolam followed by an intravenous injection of 3 g heroin, although the method of alprazolam administration remained unclear. On the note, the grandson wrote that breathing stopped approximately 1 minute after the heroin injection. Following a suicide attempt by throat slashing using the razor blade that was discovered in the flat, the grandson contacted the police for assistance and confessed to the homicide of his grandmother. He, a person who abused drugs, employed as a security guard at a local hospital, claimed to have deeply loved his grandmother and asserted that the homicide was a mercy killing per her request to end her suffering from chronic conditions.

### 2.2 Autopsy and histology findings

The body was promptly cooled to 4°C in the morgue within 2 h following the external postmortem examination conducted at the scene. The autopsy was carried out the subsequent day. During the autopsy, the absence of rigor mortis was noted in the joints. Drug intoxication was strongly suspected as the primary cause of death, as no alternative cause of death was evident upon autopsy examination. Relevant incidental findings during gross examination included signs of chronic heart failure, severe arteriosclerosis with organized thrombotic deposits within an aneurysmal dilation of the aortic arch, chronic obstructive pulmonary disease, chronic kidney injury, and breast cancer‐suspect tissue affecting the right mammary gland. No signs of metastases were noted. The injured left forearm showed vital bleedings in the subcutaneous layers but no muscle or vascular injury, histology showed initial perivascular infiltration with neutrophil granulocytes surrounding the bleeding zones. No further injuries were detected. Histological analysis of the breast tissue revealed an invasive ductal carcinoma.

### 2.3 Toxicological analyses

A full‐scale “general unknown” toxicological investigation of all available specimens was performed using immunological methods as well as GC–MS and LC–MS/MS methods. All methods have been validated according to the current guidelines of the Society for Toxicological and Forensic Chemistry [5] and have been applied in routine analysis for several years. Regular external quality control is performed by periodical proficiency testing. A detailed description of the LC–MS/MS method for quantitative determination of morphine is described elsewhere [6]. The LC–MS/MS method for quantitative determination of bisoprolol was described before [7]. The limits of quantitation (LOQs) of the detected substances in blood are 0.0011 mg/L (cocaine), 0.00089 mg/L (benzoylecgonine), 0.00066 mg/L (methylecgonine), 0.0011 mg/L (6‐monacetylmorphine), 0.001 mg/L (morphine), 0.0014 mg/L (codeine), 0.0011 mg/L (alprazolam), 0.0013 mg/L (hydroxyalprazolam), and 0.00083 mg/L (bisoprolol). If the measured values were above the calibration range, the measurement was repeated with a diluted blood sample. For the quantitative analysis in hair, two calibration ranges are used in routine (0.04–0.28 ng/mg and 0.28–2.0 ng/mg). The LOQs of the detected substances in hair are 0.032 ng/mg (cocaine), 0.061 ng/mg (benzoylecgonine), 0.032 ng/mg (6‐monacetylmorphine), 0.055 ng/mg (morphine), 0.016 ng/mg (alprazolam), and 0.025 ng/mg (hydroxyalprazolam). Results of the analysis of relevant substances in urine and stomach content as well as drug levels in venous (femoral) blood and hair are detailed in Table 1.

**TABLE 1:** Results of the toxicological analyses in various samples.

Substance	Venous blood	Urine	Stomach	Hair
6‐Monoacetylmorphine	0.028 mg/L		x	1.3 ng/mg
Morphine	1.7 mg/L	x		0.46 ng/mg
Codeine	0.10 mg/L	x	x	
Noscapine		x		
Caffeine		x	x	
Paracetamol		x	x	
Alprazolam	0.020 mg/L	x	x^a^	n.d.
Hydroxyalprazolam	n.d.			n.d.
Cocaine	0.021 mg/L	x		Approx. 41 ng/mg^b^
Benzoylecgonine	0.029 mg/L	x		1.3 ng/mg
Methylecgonine	0.035 mg/L			
Bisoprolol	0.024 mg/L	x		
Ramipril		x		

Abbreviations: n.d., not detected; x, qualitative detection.

^a^ Low signal, unsure if ingested orally.

^b^ Value extrapolated, above the calibrated range.

## 3 DISCUSSION

Active euthanasia through injections has a significant historical precedent, with a wide range of substances having been utilized. In 1924, British physician Sir F.G. Chandler documented his use of a combination of ingested and subcutaneously administered cocaine to alleviate the suffering of patients in the terminal stages of tuberculosis and other prolonged illnesses [8]. Additionally, in 1936, the severely ill British King George V was euthanized by his physician through intravenous administration of morphine and cocaine [9]. Importantly, homicides by injections administered through physicians are reported in literature. The British physician Harold Shipman killed at least 15 patients through injections of heroin or morphine. However, based on an expert witness report, the estimated number of victims was stated to be between 236 and 345 [10]. From a medical standpoint, the external postmortem investigation remains a pivotal tool in uncovering homicides [11, 12] and suicides [13, 14] through injection of drugs. Firstly, this discussion addresses the substances discovered in various body tissues in the presented case, followed by highlighting notable considerations for forensic investigators.

### 3.1 Cause of death: Acute heroin intoxication

The toxicological analysis results provided evidence of acute heroin intoxication, corroborating the grandson's claim. The blood concentration of morphine, an active metabolite of heroin, was notably elevated. Elevated blood concentrations of morphine can induce respiratory depression, leading to cerebral hypoxemia [15]. Additionally, the presence of 6‐monoacetylmorphine in the blood suggests a short survival time following heroin administration, as it is rapidly metabolized to morphine. Codeine and noscapine are contaminants commonly found in raw opium. Caffeine and paracetamol are frequently used as adulterants to dilute both heroin and cocaine [16]. However, their presence may not necessarily be related to previous consumption and / or administration of the two illicit drugs.

Benzodiazepines such as alprazolam are known to enhance the rewarding effects of heroin [17, 18, 19, 20]. Concurrent use of benzodiazepines allows for lower heroin doses to achieve a similar euphoric effect, making it economically appealing [19]. Therefore, benzodiazepines are frequently used as extenders on the black market. Moreover, alprazolam reduces anxiety, which is appreciated in an extraordinary situation such as the injection of a lethal drug dose. In the presented case, the low signal for alprazolam detected in the stomach content does not definitively indicate whether it was orally ingested. The fact that alprazolam was detected in blood but was absent in hair and its metabolite hydroxyalprazolam was neither detected in blood nor hair indicates that the woman survived the alprazolam administration only by minutes at most. The concentration of alprazolam in the blood was within the therapeutic range, typically between 0.005 and 0.050 mg/L [21]. Furthermore, deaths solely attributed to alprazolam are rare due to the well‐known plateau effect of benzodiazepines, with its abuse commonly associated with polydrug use patterns [22]. Laboratory rat experiments investigating the effects of an alprazolam‐heroin combination revealed a synergistic effect between the two substances, which may have hastened death in the presented case [17].

In a recent epidemiological study from Jeddah, Saudi Arabia, all 97 heroin‐related deaths between January 2008 and July 2018 were re‐analyzed [23]. All five heroin‐related homicide cases were also tested positive for cannabis, cocaine, tramadol, and alprazolam, whereas heroin‐related suicides predominantly involved heroin alone [23]. A low concentration of cocaine in the blood indicates antemortem consumption in the presented case. The high concentration of cocaine in hair samples may be attributed to significant cocaine consumption within the 3 months preceding death [24]. Bisoprolol and ramipril were interpreted as prescribed antihypertensive medications unrelated to the ultimate cause of death.

### 3.2 Forensic appreciation of the presented case

The case presented depicts a mercy killing wherein a geriatric woman with multiple chronic conditions including malignant breast cancer was lethally intoxicated with heroin by her grandson. None of the diseases detected during the postmortem investigations is considered life‐threatening or as a competing cause of death under the given circumstances. Given the grandson's confession and the presence of a credible farewell letter, the investigative aspect of the case was straightforward. However, had there been no confession or farewell letter, the superficial T‐shaped cut on the left forearm would have raised suspicion for self‐harm during the external postmortem examination. The T‐shaped cut on the left forearm could have been inflicted by the grandson postmortem. Regarding this, the discovery of the razor blade away from the body could be a sign that the grandson tried to make it look like a suicide after he killed the grandmother through heroin injection. However, the grandson used the razor blade for his suicide attempt and, therefore, could also have removed it from the body after he or the grandmother herself cut the arm. Moreover, the partial bleeding into the wound edges suggests that the grandmother was still alive when the injury was inflicted – conscious or unconscious. Therefore, it remains unclear to the authors who inflicted the injury under what circumstances and no further information from court are available in this regard.

An important forensic consideration arises regarding whether the case would have been autopsied solely based on the injection site, especially if any blood discharge had been cleaned before examination. Minor bruising at injection sites, particularly around the forearm and hand veins, is commonly encountered during external postmortem investigations. In elderly individuals with multiple chronic illnesses who are discovered deceased in their beds, such bruising may initially be attributed to recent medical procedures rather than homicidal drug injections. Toxicological analyses are commonly performed if an autopsy takes place based on the results of the entire criminal investigation including the external postmortems. In Hamburg, toxicological analyses are routinely performed if ordered by the public prosecutor's office in suspicious cases and each homicide case. Theoretically, if it is not classified to be a suspicious or homicide case and an additional toxicological analysis is not ordered by the public prosecutor's office and / or autopsy results in a convincing cause of death being unsuspicious regarding a potential drug overdose, intoxications could be missed. Given the high prevalence of multi‐morbidity among the elderly and the possibility that their deaths may not be unexpected, this age group is particularly vulnerable to becoming victims of homicide. A recent study from Australia analyzing homicides among individuals aged 65 years and older between 2001 and 2015 revealed several common findings [25]. Notably, victims were typically younger than 75 years (median age 72 years) and had at least one physical illness, and perpetrators had a history of illicit drug or alcohol use, diagnosed mental illness, and exposure to violence. Furthermore, the relationship between the deceased and the perpetrator tended to be intimate or familial [25]. Interestingly, all known details of the presented case align with these findings.

Individuals with a history of drug addiction, such as the grandson in this case, not only have access to relevant substances but also possess considerable knowledge of their administration. Thus, it is not surprising that intravenous injections are precisely targeted, often requiring only a single puncture even in challenging vein status, such as those in the elderly. In this case, the grandson likely had easy access to high‐quality consumables, such as the butterfly needles and scalpel found at the scene, through his employment as a security guard at a local hospital. The court trial resulted in the acquittal of the grandson as he was found to be in an exceptional mental state during the crime and his life circumstances were described to be tragic.

## 4 CONCLUSIONS

Indeed, multi‐morbid elderly individuals constitute a vulnerable demographic group susceptible to becoming victims of homicide. Therefore, meticulous documentation of injection bruises is paramount to avoid overlooking cases of consensual homicides involving injections (“voluntary active euthanasia”). Elderly individuals who have close family members with a history of illicit drug abuse may be especially at risk.

## CONFLICT OF INTEREST STATEMENT

The authors declare that they have no known competing financial interests or personal relationships that could have appeared to influence the work reported in this case report.
